# Random Functions as Data Compressors for Machine Learning of Molecular Processes

**DOI:** 10.1021/acs.jctc.5c01638

**Published:** 2026-01-29

**Authors:** Jayashrita Debnath, Gerhard Hummer

**Affiliations:** † Department of Theoretical Biophysics, 28273 Max Planck Institute of Biophysics, 60438 Frankfurt am Main, Germany; ‡ Institute of Biophysics, Goethe University Frankfurt, 60438 Frankfurt am Main, Germany

## Abstract

Machine learning (ML) is rapidly transforming the way molecular dynamics simulations are performed and analyzed from materials modeling to studies of protein folding and function. ML algorithms are often employed to learn low-dimensional representations of conformational landscapes and cluster trajectories into relevant metastable states. Most of these algorithms require the selection of a small number of features that describe the problem of interest. Although deep neural networks can tackle large numbers of input features, the training costs increase with input size, which makes the selection of a subset of features mandatory for most problems of practical interest. Here, we show that random nonlinear projections can be used to compress large feature spaces and make computations faster without a substantial loss of information. We describe an efficient way to produce random projections and then exemplify the general procedure for protein folding. For our test cases NTL9 and the double-norleucin variant of the villin headpiece, we find that random compression retains the core static and dynamic information of the original high-dimensional feature space, making trajectory analysis more robust.

## Graphical abstract

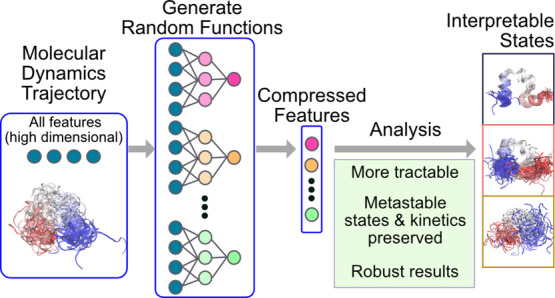

## 1 Introduction

Molecular dynamics (MD) simulations have proven to be very useful tools for the study of biomolecular systems. Large systems with millions of atoms are now frequently simulated for microseconds to milliseconds.^1–3^ This remarkable progress has, however, led to a new challenge: the problem of analyzing long trajectories of high-dimensional data.^4^

Although the dimensionality of each frame of an MD trajectory scales with the number of particles in the box, this dimensionality of the trajectories can be reduced due to the inherent time scale separation of the encoded dynamics.^5–7^ For instance, ions and water relax much faster than protein conformations. The dynamics of the protein can be separated into the slower global movements of domains and faster fluctuations in the flexible regions. These observations have motivated researchers to eliminate degrees of freedom considered fast and nonessential. Such elimination typically begins with ignoring the solvent degrees of freedom. Following this step, it has been a common practice to project the dynamics of the trajectory onto a few collective variables (or order parameters) such as the root-mean-square deviation (RMSD) from a structure, dihedral angles, or distances of interest. However, with the increasing size and complexity of the systems, simplistic elimination strategies no longer work well, not least because the slow degrees of freedom become less obvious. Consequently, machine learning techniques are routinely employed to reduce the dimensionality of MD trajectories.

Machine learning based approaches not only help in learning meaningful lower dimensional representations of trajectories, they also help in clustering trajectories into metastable states or learning collective variables for enhanced sampling methods.^4,8–12^ In practical applications, one usually starts by choosing a set of input features for training the model. In systems like proteins in a box of water, the water molecules are often neglected and trajectories are represented using internal coordinates of the protein atoms such as Cα distances (or contacts), dihedral angles of the residues, or Cartesian coordinates of a subset of atoms. The goal of any dimensionality reduction technique then is to find an *n-*dimensional map Φ of the original *N-*dimensional feature space, 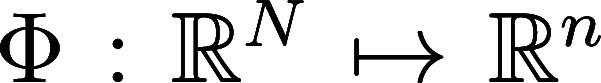 where *n* ≪ *N*, which resolves relevant states and is associated with a simple, near-Markovian dynamics. Linear maps are represented by a *n* × *N* matrix M . Many strategies have been employed over the years for generating such mappings.^13^ One of the most popular algorithms used for MD trajectory data is principal component analysis (PCA),^14,15^ in which a linear combination of the initial feature space is obtained in a way that optimally describes the variance of the data. Another dimensionality reduction technique that is often employed in the context of time dependent data is time-structure independent component analysis (TICA) where the data are projected onto the generalized eigenvectors of time-lagged covariance matrix, accounting for static correlations, to separate slow from fast relaxation processes^16^ that generated it. While PCA, TICA, or other approaches like linear discriminant analysis (LDA) often result in meaningful lower dimensional representations of the data, these linear methods often fail to provide meaningful lower dimensional representation of the data when the dimensionality of input feature space is very high.^17^

For (bio)­molecular systems, agnostic feature spaces are large. The number of pair distances scales quadratically with the number of residues in a protein and the number of dihedral angles scales linearly. Such features thus cannot be used directly as input for most linear machine learning algorithms, and a reduction of dimensions becomes necessary even before the application of these techniques. Some nonlinear dimensionality reduction techniques such as t-distributed stochastic neighbor embedding (t-SNE),^18^ Kernel PCAs, self-organizing maps,^19^ Isomaps,^20^ Sketch map,^21^ EncoderMap,^22^ VAMPnet^23^ are increasingly being employed for analyzing MD simulations as they can deal with input of higher dimension.^8^ However, these methods too cannot deal with excessively large input dimensionality, as would be the case when solvent degrees of motion are included or when proteins are not small. In such cases, discarding some input features is imperative, even when working with nonlinear models for dimensional reduction.

Recently, a lot of work has focused on extracting a subset of useful features from large data sets to minimize input size.^24–29^ AMINO^25^ and MOSAIC^24^ use mutual information to identify and remove redundant features on the basis of *N* × *N* covariance matrices. Reweighted diffusion maps^27^ and Spectral oASIS^28^ solve for their eigenvalues and eigenvectors, and then select optimal subsets of features that, respectively, maximize the spectral gap and best reconstruct the leading eigenvectors. Although these methods can be very effective at removing inessential features, they tend to become computationally expensive for large data sets.

Here, we propose an alternative approach to reducing the size of the set of input features. In contrast to the existing methodologies, we compress the large feature sets using random nonlinear projections without specifically eliminating particular input features. The compressed feature sets are then used as input together with established analysis tools, such as the ones discussed above, for which the use of the full feature set would have been challenging or impossible. By repeating this procedure over different random projections, we effectively establish low dimensional representations of the conformation space that capture the slow kinetics of conformational transitions. Random projections are thus an efficient strategy to produce compressed feature sets before the application of any machine learning algorithm for analyzing molecular dynamics trajectories. In the following sections, we introduce random projections, propose a way to generate random nonlinear projections for MD trajectory data and demonstrate that such compressed feature sets preserve essential properties of the original high dimensional data for protein-folding studies.

## 2 Methods

Our strategy to generate random nonlinear projections is inspired by the Transition Manifolds method^7,30^ and the Whitney Embedding Theorem,^31^ two approaches that together guarantee the existence of a lower dimensional embedding for MD trajectory data. Mathematically, our random nonlinear projection approach can be seen as an extension of the random mappings method, a linear dimensionality-reduction technique that was initially proposed and applied in the context of document classification. In the random mappings method, a lower dimensional map is generated using a *n* × *N* matrix M that is randomly initialized. A linear random mapping is given by $x_{n\times T}=M_{n\times N}X_{N\times T}$ , where ***X*** is a matrix of dimensions *N* × *T* with *N* the number of input features and *T* the length of the trajectory data, and *x* is a *n* × *T* matrix representing the input data mapped into a space of dimension *n* (*n* ≤ *N*).^32^

If the column vectors of the random matrix $M_{n\times N}$ are drawn from a mean-free, unit-variance distribution, the resulting random combinations of the original high-dimensional features are almost orthogonal.^33–35^ Additionally, when the lower dimension is sufficiently large these mappings preserve well all the pairwise distances between the original data (Johnson–Lindenstraus lemma^36^). Reducing the dimensionality using these mappings can therefore speed up classification or clustering tasks while causing almost no loss of information as long as the embedding dimension *n* is sufficiently large.^32,37^ However, the required dimension *n* is extremely large for typical MD trajectory data. Many other strategies have thus been proposed for generating random projections,^38,39^ including approaches to generate random nonlinear projections.^40^ In the following paragraphs, we propose our strategy to generate random nonlinear projections for MD trajectory data that fall into the same category as the latter approaches.

### 2.1 Constructing Random Projections of MD Data

To generate compressed feature spaces, we perform a forward propagation of our high-dimensional trajectory data through randomly initialized feed forward networks. Generating these projections using a single-layer perceptron would be mathematically equivalent to the linear random mapping method due to the absence of any nonlinear activation. However, the final projected space generated using multilayer perceptrons with nonlinear activations is nonlinear even in the absence of any bias. An *n*-dimensional nonlinear embedding can therefore be generated either using *n* different multilayered networks, each with a single output neuron or from a single multilayered network with *n* output neurons (see Supporting Information (SI) Figure S1).

Networks with one output have an architecture as shown in Figure 1, with a random number of hidden layers and a random width for each of the hidden layers. Mathematically, such a transformation is equivalent to

$$g_{\alpha }^{\left(0\right)}(X)=W_{\alpha }^{\left(0\right)}X+B_{\alpha }^{\left(0\right)}$$ 1

$$g_{\alpha }^{\left(1\right)}(X)=\varphi_{\alpha }^{\left(1\right)}(W_{\alpha }^{\left(1\right)}g_{\alpha }^{\left(0\right)}(X)+B_{\alpha }^{\left(1\right)})$$ 2

$$g_{\alpha }^{\left(h_{\alpha }-1\right)}(X)=\varphi_{\alpha }^{\left(1\right)}(W_{\alpha }^{\left(h_{\alpha }-1\right)}g_{\alpha }^{\left(h_{\alpha }-2\right)}(X)+B_{\alpha }^{\left(h_{\alpha }-1\right)})$$ 3

$$g_{\alpha }^{\left(h_{\alpha }\right)}(X)=W_{\alpha }^{\left(h_{\alpha }\right)}g_{\alpha }^{\left(h_{\alpha }-1\right)}(X)+B_{\alpha }^{\left(h_{\alpha }\right)}$$ 4

where ***W***_α_^(*i*)^ ***X***, *B*_α_^(*i*)^ are weights and biases of the *i* ^*th*^ layer, *g*_α_^(*h*_α_)^(***X***) is a one-dimensional vector obtained using *h*_α_ hidden layers activated by ELU activations, φ_α_^(*i*)^, after each hidden layer *i* for a given network α. The outputs *g*_α_^(*h*_α_)^(***X***) are standardized using min-max normalization. An *n-*dimensional random nonlinear projection, *g* ^*n*^(***X***), is then obtained by generating *n* random function vectors {*g*_1_^(*h*_1_)^(***X***), *g*_2_^(*h*_2_)^(***X***),..., *g*_*n*_^(*h*_*n*_)^(***X***)}. As these networks are not trained, the method used for initializing the weights and biases influences the quality and stability of the projections obtained. In the following sections, we have used the Xavier initialization scheme for initializing the weights of the networks while the values of the biases were initialized from a uniform distribution.

**Figure 1:**
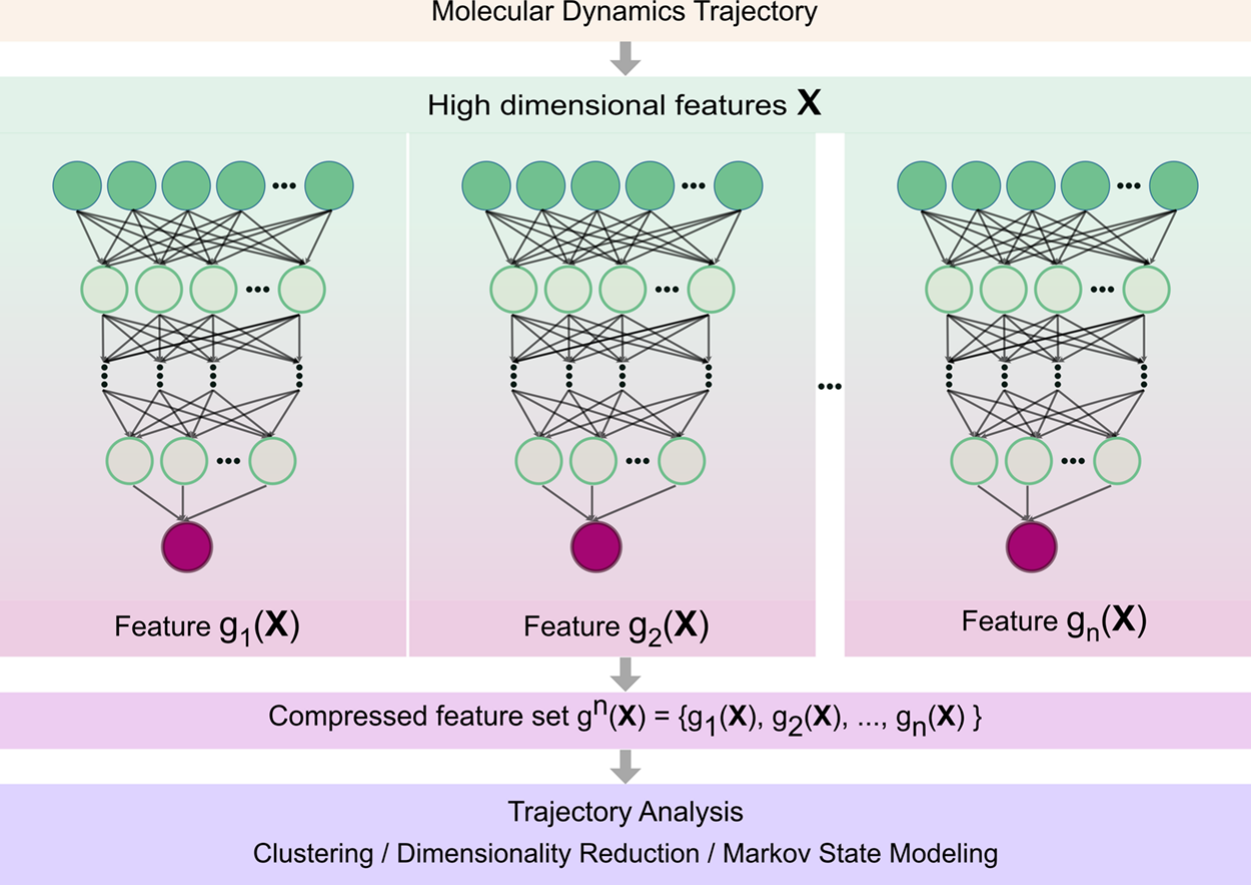
Random compression of the MD trajectories. Vectors ***X*** containing *N* molecular dynamics features of the structures along a MD trajectory are compressed to dimension *n* ≪ *N* using different random networks *g*_*i*_(***X***) with *i* = 1, ···, *n*. The resulting *n* low-dimensional projections are then used for further trajectory analysis.

A good compressed feature set should ideally include a diverse set of features that are not highly correlated. In practice, using a single network often results in many correlated functions as output. However, when multiple networks having the same or different architectures (varying the number and depth of the hidden layers) are used to generate different one-dimensional embeddings, the resulting random functions are often less correlated. In the SI text and SI Figures S2–S4, we have provided a more detailed comparison of results obtained with different methods for compressions. In the following sections, we restrict our discussion to compressed feature spaces that are generated by using multiple independent networks.

As discussed earlier, many artificial neural network-based methods have been developed recently to learn reaction coordinates or committors from MD trajectories.^4,41–46^ Here, we intend to compress the high-dimensional space to make any further analysis more tractable. The best lower dimensional representation or reaction coordinate might not be obtained in this process. However, the compressed space, having a higher dimensionality than the best lower dimensional representation, should still be able to retain all relevant kinetic and metastable state information in order to be effective. Having proposed a way to generate compressed feature spaces, we assess their ability to retain time scales and clusters for different systems in the following sections.

## 3 Results

### 3.1 Alanine Dipeptide

Alanine dipeptide in aqueous solution at ambient temperature and pressure is an extremely well-studied system whose dynamics is known to be captured almost entirely by the two Ramachandran angles (φ, ψ). Here, we applied random projections to three independent trajectories of alanine dipeptide in TIP3P water at 300 K, each 250 ns long^47^ and available in the public repository mdshare (https://markovmodel.github.io/mdshare/). As TICA is often used for analyzing MD simulations, we show in Figure 2 how well the TICA components are reproduced if compressed features are used as input for these methods instead of all 45 heavy atom distances of the molecule. As randomly compressed feature sets are not unique, we generated 25 different sets of features of different dimensions, taking all the distances as input for the random function generator. We then obtained TICA decompositions using these compressed features as an input. In order to evaluate the quality of these decompositions, we analyzed the distributions of the first five eigenvalues over 25 trials (see Figure 2a). We found that a lower-dimensional compressed space was sufficient to reproduce the first TICA component, while larger dimensions were necessary for the subsequent components. With only a small set of random functions, components 2 and 3 were mixed, as were components 4 and 5, in both cases, giving the smaller of the two eigenvalues (Figure 2a). With sufficiently many compressed features, we obtain similar TICA eigenvalues and projections onto the TICA components (Figure 2c). Even though TICA is a fairly simple linear decomposition method, it can be seen that nonlinearly compressed feature sets having dimensions less than half the dimensions of the original feature set can capture both the variance and the underlying dynamics of the data set. As a nonlinear method, VAMPnet^23^ improves upon many limitations of TICA and can be used to obtain both relaxation time scales and clusters from MD trajectories. In the SI Text and SI Figures S1, we show the time scales obtained from VAMPnet using PCA components as input. In SI Figures S3 and S4, we show that fewer than 10 compressed features fed into VAMPnet are sufficient to capture all the slow modes of this system when compressed features are used, created either using a single multilayered network or multiple networks.

**Figure 2:**
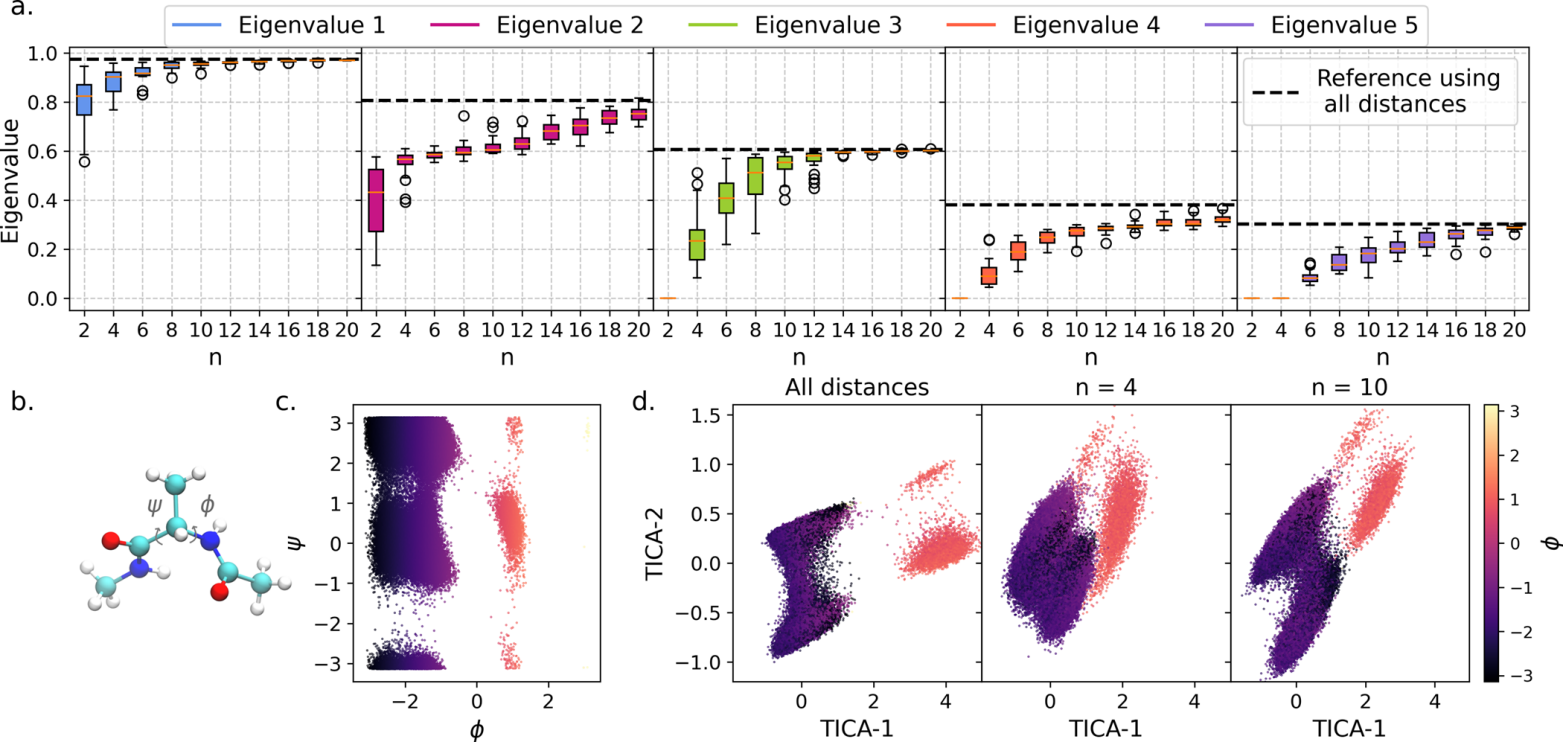
Alanine dipeptide results: (a) Eigenvalues of the 5 slowest TICA components (lag = 1 ps) obtained using different numbers of random features as input (25 trials). (b) Alanine dipeptide molecule showing the φ and ψ dihedral angles. (c) Scatter plot of the dihedral angles showing the different states. (d) Examples of TICA projections obtained using different input features: all 45 distances (left), compressed dimension *n* = 4 (center), and compressed dimension *n* = 10 (right). The scatter plots in parts c and d have been colored according to the φ coordinate to highlight the separation of states.

### 3.2 NTL9

Recently, Mardt et al.^23^ developed a neural-network based approach, VAMPnet, to extract kinetic models from high-dimensional trajectory data. VAMPnet intended to replace the multistep approach required to obtain Markov State Models (MSM). It uses a Siamese network like architecture, with two lobes that share weights, to describe the time lagged data, at user-specified time lag (τ). Given a user-specified number of states, VAMPnet attempts to resolve the data into different processes by optimizing the variational approach to Markov process (VAMP) scores. In their paper, Mardt et al.^23^ used 666 nearest-neighbor atom contacts defined using *c*_*ij*_ = exp­(−*d*_*ij*_/*d*_0_) as input to VAMPnet, with *d*_*ij*_ the pair distances and *d*_0_ a characteristic length, and obtained 2-state and 5-state decompositions of the trajectory of NTL9, a 39-residue protein whose folding dynamics was simulated for about 1.11 ms by Lindorff-Larsen et al.^48^ Here, we evaluate if compressed features can produce accurate time scales and state decomposition when used as input for VAMPnet.

In Figure 3, we show the time scales and states obtained by training VAMPnets with all 6786 backbone contacts and the ones obtained using different numbers of compressed features. The compressed features were obtained from randomly chosen architectures having a randomly chosen depth between 5 and 20 layers and each layer having a random width between 2 and the input dimension. The time scales and states reported in the figure were obtained from 50 trials for each case, and new random functions were generated for each trial, which then used the new compressed features as input. VAMPnets were trained using a fixed time lag of τ = 50 ns. However, for estimates of the relaxation time, the lagtime was varied but with the cluster assignment fixed to that of lagtime τ. The architecture of the VAMPnet lobes varied depending on the number of input features, and the size of the network increased with increasing input size. More details on the architecture of the VAMPnets are provided in the SI Text and SI Table S1.

**Figure 3:**
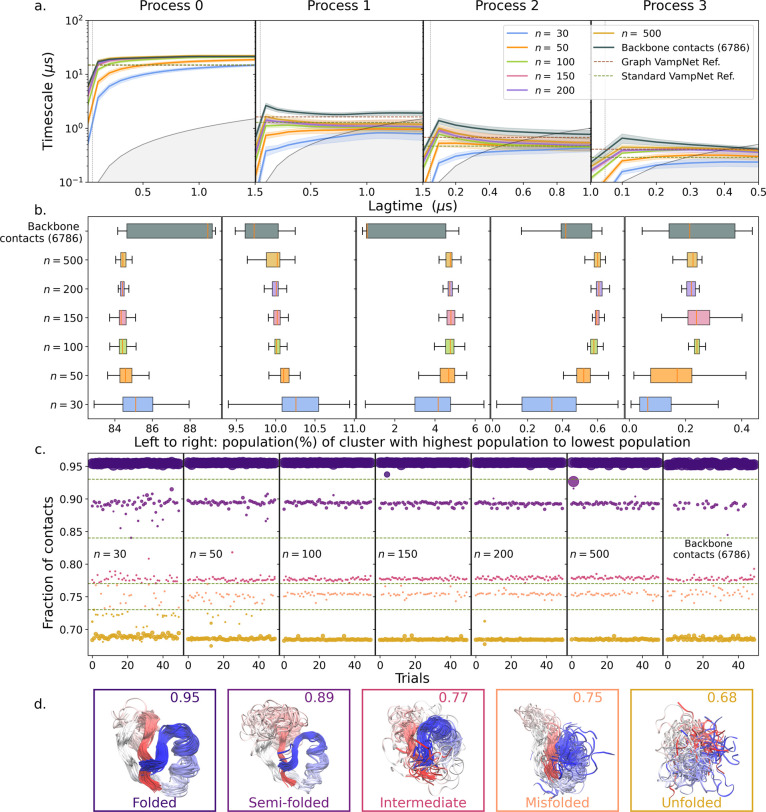
Random compression applied to folding of NTL9 protein: (a) time scale of 4 slowest relaxation processes (left to right) extracted from 50 trials as a function of lagtime, with fixed time lag of 50 ns for VAMPnet training (vertical dotted line). The dimensions of the random projections, when used, are indicated by *n* in the legend. Backbone contacts (6786) indicate that no compression is used. “Graph VAMPnet” are the results of Ghorbani et al.,^50^ and “Standard VAMPnet” those of Mardt et al.^23^ Error bars indicate the standard deviations across the different trials. The gray area indicates time scales less than the lagtime. (b) Population of the five clusters obtained. Left to right: most populated cluster in each trial to least populated. (c) Mean fraction of native contacts for the five clusters obtained in the 50 trials for each method, as indicated in each subpanel. Colors correspond to the clusters in part d. The size of each dot is proportional to the cluster population. The four dashed lines at contact fractions of 0.93, 0.84, 0.77, and 0.73 indicate boundaries between different cluster structures. (d) Backbone structures representative of the clusters with different fractions of native contacts in c. The clustering was obtained in one of trials with *n* = 100 random projections. The colors of the surrounding boxes correspond to the color of the cluster in c. The value of the mean fraction of native contacts in each cluster is shown at the top for reference.

We find that random projections of dimension *n* ≥ 100 capture the slowest relaxation process of NTL9 with about the same characteristic relaxation time as obtained by using all 6786 backbone contacts (Figure 3a). The faster relaxation processes from the VAMPnets with all backbone contacts are somewhat slower than those from random projections, albeit with a more pronounced lagtime dependence. To gain a deeper understanding, we looked at the clusters produced by VAMPnets as representatives of the kinetic states. At first, we compared the similarity scores using popular metrics to evaluate the consistency of the clustering across different methods (see SI Figure S5). However, due to significant differences in the populations of the clusters, these metrics did not work very well. We therefore looked at the cluster population distributions and the mean fraction of native contacts for clusters.^49^

Figure 3b,c shows the cluster population and the mean fraction of native contacts for each cluster obtained in each trial, respectively. In Figure 3b, the cluster population distributions for each trial are shown using the box plots, where the boxes represent the interquartile range, while the median, minimum, and maximum values are shown using orange lines and whiskers, respectively. In Figure 3c, 5 dots represent the 5 states that were obtained for each trial using different input features. The size of these dots scales with the population of the cluster. For a given set of input feature types, the clustering outputs can be considered as consistent when all 5 dots have consistent sizes and mean fraction of contacts for multiple trials. For random projections of dimension *n* ≥ 100, we find that across the respective set of 50 trials the clusters are consistent with each other, both in terms of their population (Figure 3b) and the extent of native structure in them (Figure 3c). By contrast, when using all backbone contacts in VAMPnet trials, the variation between the resulting 50 clusters is large (Figure 3b (top) and Figure 3c (right)). We note that the five clusters correspond to the folded state, the unfolded state, and three folding intermediates (Figure 3c), and visually agree with the states reported by Mardt et al.,^23^ also in terms of the populations.

For NTL9, the use of high-dimensional input results in a larger variation of the resulting dimensionality reduction maps in repeated trials, which may offset the finer resolution of the conformational dynamics. When all 6786 backbone contacts are used as input for VAMPnet, the populations of the clusters are distributed over a wider range of values and structures are often misclassified (Figure 3b,c). Furthermore, the network fails to find the third most populous semifolded state in many trials, and multiple misfolded or unfolded clusters are found having mean fraction of native contacts between 0.73 and 0.81. By contrast, using compressed features results in a more consistent clustering as the populations of the different clusters and the mean fraction of native contacts are consistent not only across different trials but also across different dimensionality of compressed spaces.

Even a comparably small number of compressed features resolves the dominant processes. Although both the time scales and states are not very accurate with a compressed dimension of *n* = 30, it was possible to obtain the highly populated folded and unfolded states even for this case. However, the other three states are often misclassified, as is evident from the scattered points in the mean fraction of native contacts between 0.70 and 0.80. This should also explain the significantly shorter relaxation times obtained with *n* = 30. However, as the dimensionality of compressed space is increased, the clustering tends to be more consistent and the relaxation times converge. As few as *n* = 100 compressed features were sufficient to obtain also accurate time scales and populations (Figure 3a). The dimension of the compressed space (*n* = 100) is significantly smaller than that of the original feature set (6786) or the set of 666 features used by Mardt et al.,^23^ making any analysis significantly less computationally expensive and more efficient.

In SI Figure S6, we show the results obtained when VAMPnet architectures are scaled with increasing input dimensions. We find that using larger networks has a minimal impact on the quality or consistency of the clusters. In SI Figure S7, we show the results obtained when a randomly selected set of contacts is used as input to VAMPnet instead of randomly compressed features. We find that random feature selection neither improves nor worsens the clustering results. By contrast, using compressed features significantly improves the consistency of clusters. In SI Figure S8, we also show, for reference, the results obtained using TICA and PCA. Overall, we conclude that for the NTL9 trajectory, a low-dimensional compression retains the static and dynamic information encoded in the higher dimensional trajectory.

### 3.3 Double-Norleucin Variant of Villin Headpiece

Villin headpiece subdomain (HP35) is a fast-folding protein with 35 residues that has been used as a test case for many protein-folding studies. One particular 300 μs long simulation of the norleucine double-mutant variant (Lys24Nle/Lys29Nle) of HP35 (PDB: 2F4K) simulated at 360 K by Lindorff-Larsen et al.^48^ has been studied extensively over the years.^51–60^ While most of these works concluded that the trajectory could be clustered into 4 states: folded, partially folded, intermediate, and unfolded state, there seems to be no consensus in the literature on the exact splitting of states. For instance, Nagel et al.^54^ reported that the native basin is highly populated (≈68%), while Ghorbani et al.^50^ assigned only 22.93% population to the native folded state and 71.93% population to the unfolded state. Also, an exhaustive analysis of MSMs constructed using different input features by Nagel et al.^54^ demonstrated the necessity of feature engineering using this system. Their results indicated that selecting different types of input features, contacts, or dihedrals, influenced the number of macrostates and consequently the implied time scales for different processes. The ambiguous state splitting and complicated feature selection for this system tempted us to investigate the consistency of clusters and time scales obtained using compressed features constructed with different inputs for the random function generator.

As for NTL9, we used VAMPnets to obtain the clusters and implied time scales for the double-norleucine variant of villin, which we below refer to as “villin”. While different types of features were used as input for VAMPnets, all networks had 3 hidden layers and 4 output neurons (4 states). Further details on VAMPnets can be found in the SI Text and SI Table S2. We used 1.5 ×10^5^ frames of the 300 μs trajectory and a time lag of τ = 20 ns. We investigated the states obtained with 8 different sets of input features for VAMPnet: all backbone contacts (5460), all Cα contacts (595), all dihedral angles (66), all positions (1731), and compressed features obtained using each of these 4 types of features as input to the random function generator. For the experiments with dihedral angles as input, we used the shifted dihedrals provided by Nagel et al.^54^ and for the cases with positions as input, we aligned the backbone atoms in the trajectory to those in the folded structure for all 577 atoms in villin. In Figure 4, we have summarized the VAMPnet results obtained in 25 trials for each input feature type, while we have provided more detailed results in the SI Figures S9–S11.

**Figure 4:**
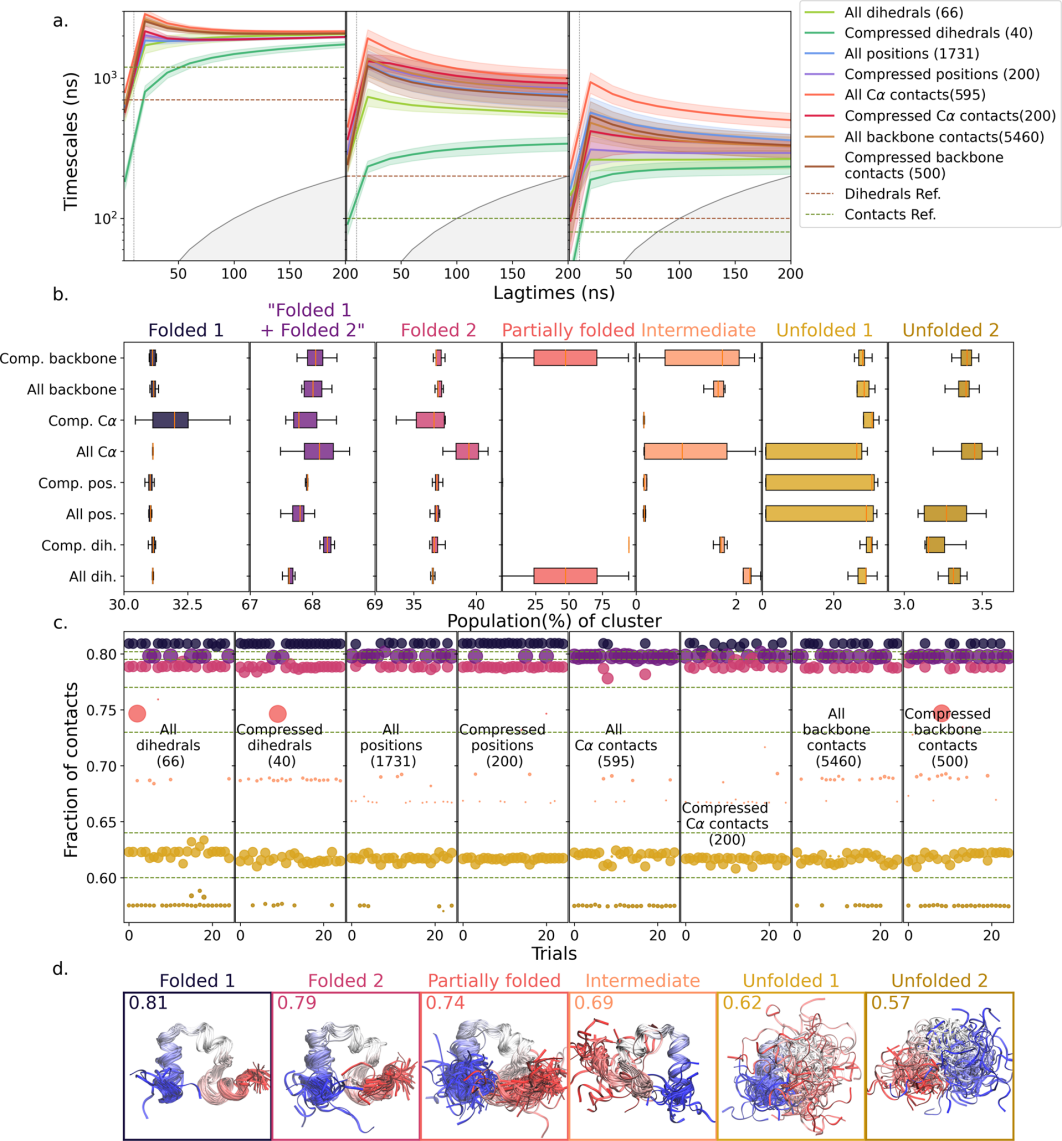
Random compression applied to folding of villin headpiece: (a) time scale of three slowest relaxation processes (left to right) extracted from 25 trials as a function of lagtime, with fixed lagtime of 20 ns for VAMPnet training (vertical dotted line). The features used, the dimension of the random projections and the method are indicated in the legend. “Dihedrals Ref.” and “Contacts Ref.” are the reference results from Nagel et al.^52^ Error bars indicate standard deviations across the different trials. The gray area indicates time scales less than the lagtime. (b) Population of the seven clusters obtained. Clusters were grouped according to their mean number of native contacts shown in part c. (c) Mean fraction of native contacts for the clusters obtained in the 25 trials for each method, as indicated in the figure. Colors correspond to the clusters in d. The size of each dot is proportional to cluster population. The six horizontal dashed lines at contact fractions of 0.802, 0.795, 0.77, 0.73, 0.64, and 0.6 indicate boundaries between different cluster structures. (d) Backbone structures representative of the clusters with different fractions of native contacts in panel c. Shown are randomly chosen representatives of each cluster across trials and methods. The colors of the surrounding boxes correspond to the color of the cluster in panel c. The value of the mean fraction of native contacts in each cluster is shown on top for reference.

In Figure 4a, we show the three slowest relaxation times obtained using different feature types. It is encouraging to note that the slowest time scales obtained in all our trials converge to similar values. This indicates that the folded–unfolded transition was correctly distinguished in all of the cases. The differences between the different feature sets are more pronounced for the faster time scales, indicating differences in folding intermediates identified by different approaches. In all cases, however, the time scales obtained using either complete feature sets or compressed features as input to VAMPnet are much slower than the best time scales reported in the literature.^54^ According to the Rayleigh Variational Principle,^61,62^ our models are thus at least on par with those of earlier studies in resolving the Markov states. Additionally, the time scales obtained using contacts and positions are consistent with each other. By contrast, the slowest and second slowest time scales obtained using dihedral angles are somewhat faster. It is also only in the case of dihedral angles that the time scales obtained using compressed features converge to a much lower value than using the complete set of features. We conclude that positions and contacts better resolve the dynamics here than dihedrals.

To gain a structural understanding and shed light on the variations between methods, we examined the mean fraction of native contacts for each of the 4 clusters obtained across the 25 trials for different inputs. To our surprise, we observed a pattern for villin that was very different from what was observed for NTL9. At a first glance, we could not find any consistent 4 state split for this system using any of the input features. However, we noticed that the clusters obtained could easily be separated into 7 sets using their mean fraction of native contacts. We found that in some of our trials, the folded state was subdivided into two states (Folded1 and Folded2, the latter having slightly more disordered termini), each having a population of about 30% of the population, while in other trials a single folded state with a population of about 68% was found. This result could explain the difference in native state population observed by Nagel et al.^54^ and Ghorbani et al.^50^ Due to the very different featurization and clustering approaches in these earlier studies, it seems likely that they obtained either the merged folded state or the subdivided Folded1 and Folded2 states. In addition to these folded states, we found partially folded, intermediate, and two unfolded states in some trials. The mean time scales obtained for different processes with different input features were therefore an average of time scales over different processes. This may explain why it was not possible to obtain a consistent 4 state splitting of states using any of the input feature sets for villin.

Nevertheless, the clusters regrouped according to the contact fractions (Figure 4c) into 7 states have quite consistent structures (Figure 4d) and populations (Figure 4b,c). Our inability to consistently split the villin trajectory data, and the inconsistencies between published clusterings discussed above, could have multiple possible causes. The most obvious reason is that villin may have more than 4 states in the examined time regime. However, we could not get VAMPnet to converge with 7 states in this example. Another factor could be that the trajectory is not long enough to confidently determine the precise splitting of states. Despite the inconclusive splitting, Figure 4d demonstrates that running multiple trials with different feature sets can give a more fine-grained view of the mechanism of the folding process. Additionally, running multiple trials with compressed features promises to be a faster way to obtain different clustering solutions and gain a better idea about the number of possible states.

## 4 Conclusions

We have used neural networks for the compression of high-dimensional feature spaces of molecular dynamics trajectories. We found that random compression of the input feature spaces preserves static and dynamical information encoded in the high-dimensional trajectory. We have demonstrated that when a sufficient number of random functions are used to compress the trajectory data, the implied time scales and metastable states can be reliably extracted. Although we do not yet have a metric to estimate the minimum compressed feature dimension required to extract accurate time scales, we found here that a compression to ∼10% of the initial feature dimension led to reliable time scales and clusters using VAMPnet. Having lower dimension, states and relaxation time scales tend to be more robust compared to an analysis of the full feature space. The random features, therefore, not only reduce the need for careful feature engineering but also offer a reliable way to reduce feature space without introducing any inherent bias. The compression of feature spaces has the potential to reduce the cost of training neural network based models for machine learning applications. They become particularly useful when the high dimensionality of inputs becomes an analysis bottleneck. Interestingly, we found in our numerical trials that using *n* independent random projections tended to produce better results than extracting the *n* projections from one random network, as the use of independent projection networks minimizes correlations. Although we have focused here only on obtaining dimensional reduction and the construction of accurate Markov state models using VAMPnets, it is important to note that such compressed features could potentially be used as input for any machine learning model.

## Supplementary Material



## Data Availability

Data available at 10.5281/zenodo.17192703.

## Notes

Alanine dipeptide (SI Text 1.1); NTL9 (1.2); Villin (1.3); architecture of VAMPnet lobes for NTL9 (Table S1); varying architectures of VAMPnet lobes for NTL9 (Table S2); architecture of VAMPnet lobes for double-norleucin variant of villin headpiece (Table S3); two different approaches for obtained randomly compressed features (Figure S1); implied time scales for alanine dipeptide obtained from VAMPnet using different numbers of PCA components (Figure S2); implied time scales obtained from VAMPnet using compressed features from single network as input (Figure S3); implied time scales obtained from VAMPnet using compressed features from multiple networks as input (Figure S4); distribution of similarity scores for state assignments obtained using different VAMPnet inputs for the NTL9 protein (Figure S5); larger networks used for VAMPNet of NTL9 (Figure S6); randomly selected backbone contacts in NTL9 used as input for VAMPNet (Figure S7); TICA and PCA components obtained for NTL9 (Figure S8); implied time scales for the double-norleucin variant of villin (Figure S9); fraction of native contacts in the double-norleucin variant of villin (Figure S10); and distribution of similarity scores for state assignments obtained using different VAMPnet inputs for the Villin trajectory (Figure S11) (PDF)
