# Supplementary material for: Random Functions as Data Compressors for Machine Learning of Molecular Processes
Source: J Chem Theory Comput. 2026 Jan 29;22(3):1504–13. doi: 10.1021/acs.jctc.5c01638 (PMC12895416; doi:10.1021/acs.jctc.5c01638)
Supplement: Supplementary file 1 [file ct5c01638_si_001.pdf]

Supporting Information: Random functions as data compressors for machine learning of molecular processes

Jayashrita Debnath*,† and Gerhard Hummer*,†,‡

†Department of Theoretical Biophysics, Max Planck Institute of Biophysics, 60438 Frankfurt am Main, Germany
‡Institute of Biophysics, Goethe University Frankfurt, 60438 Frankfurt am Main, Germany

E-mail: jayashrita.debnath@biophys.mpg.de; gerhard.hummer@biophys.mpg.de

1 SI Text

1.1 Alanine dipeptide

Alanine dipeptide is a well-studied system whose dynamics can largely be characterized in terms of its two Ramachandran dihedral angles (ϕ, ψ). For the given force field, solvent, and a near-ambient thermodynamic state,[S1] we expect to find 6 metastable states, and consequently 5 timescales in Markov state modeling (MSM). Mardt et al.[S1] used the heavy atom positions (30) and interatomic distances (45) of Alanine dipeptide as input features to obtain the timescales of all 5 processes. In the following sections, we generate different types of compressed features using distances as input features and obtain a 5 state decomposition of the input trajectories. In the first case, we use different numbers of principal components as input; in the following two cases, we compress the input features using two types of random compression networks as shown in Figure S1.

1.1.1 Dimensionality reduction using principal components

For reference, we performed a principal component analysis (PCA) of the alanine dipeptide trajectories to reduce the dimensionality of the input features for VAMPnet. We use varying numbers of the top principal components ($n = 4, 6, 8, 10, 15$) as an input to VAMPnet, sorted from largest to smallest principal component. In Figure S2, we show the mean and standard deviation of the timescales obtained over 25 trials for each value of n. For $n \geq 6$, the three slowest timescales could be resolved, but not the fourth and fifth slowest timescales. As principal component analysis is a deterministic approach, there is almost no variability in the VAMPnet timescales across the 25 trials. In the following, we show results for alanine dipeptide obtained with VAMPnet and different random compressions.

1.1.2 Dimensionality reduction using random compression: multiple random functions from one network

For comparison, we generated n-dimensional compressed features by a single neural network with n outputs, similar to the case shown in Figure S1a. In Figure S3, we show the mean and standard deviation of the timescales obtained for alanine dipeptide, using compressed features of different dimensionality as input to VAMPnet across 25 trials. For each dimensionality n, we performed 25 trials, each with an independently generated random neural network to obtain a new set of compressed features. We find that the VAMPnet results show considerable variability, irrespective of input dimensionality. However, unlike with PCA inputs, VAMPnet can identify 5 distinct timescales on an average, even in the case of $n = 4$. Additionally, as the input dimensionality increases, the timescales become more consistent across different trials.

1.1.3 Dimensionality reduction using random compression: one random function from one network

To suppress possible correlations between the inputs, we then obtained random compressions by using n separate networks with one output each (see Figure S1b), as in the main text. In Figure S4, we show the mean and standard deviations of timescales obtained over 25 different trials with varying numbers of input features. As with PCA inputs and a single network for random compression, a new compressed feature set was generated for each trial. As the dimensionality of the compressed feature space increases, $n \gtrsim 6$, the timescales obtained by using random features become indistinguishable from those obtained using the full feature space.

Although PCA can be an excellent choice for dimensionality reduction when large-amplitude motions dominate the slow relaxation processes, it become impractical for large data dimensionality, as it requires calculating large matrices and their singular value decomposition or diagonalization. As shown in Figure 2 of the main text, random features can be used as an additional compression layer before applying other dimensionality reduction techniques, such as TICA or PCA. It can also be concluded from Figures S4,S3 that using multiple networks resulted in accurate and more consistent timescales than using a single network to generate all features. Therefore, we have used the approach shown in Figure S1b, to generate randomly compressed features for all the examples shown in the main text and for the remaining examples in the SI.

1.2 NTL9

We obtained relaxation timescales for NTL9 using both VAMPnet and TICA, with compressed features and all backbone contacts as input. In the following paragraphs, we provide further details on the input and results.

1.2.1 VAMPnet

VAMPnet architecture. We trained the NTL9 VAMPnet models for five states. In each of our 50 trials, we have either used all backbone contacts or compressed features created

by transforming all backbone contacts as input for VAMPnet. To ensure a more consistent comparison, we used similar VAMPnet lobes for the different sets of input parameters, except for the case of 30-dimensional compressed features. The number of hidden layers and the decrease in their size were determined by the formula suggested by Mardt et al.[S1]. The width of a hidden layer (n_l) was defined using $n_l/n_{l+1} = (n_{in}/n_{out})^{1/d}$ where d is the network depth excluding the input layer, n_{in} and n_{out} are the number of input features and states respectively. We used Exponential Linear Unit (ELU) for activation and a batch size of 10000. VAMP-2 scores were optimized during the training and 10 ns time lag was used. The input architectures for VAMPnet models have been provided in Table S1.

Cluster consistency measurements. To measure the consistency of the state assignments obtained from VAMPnet for a given type of input descriptor, we calculated four different similarity scores (Fowlkes-Mallows(FM) score[S2], Adjusted Rand Index (ARI)[S3], Normalized Mutual Information (NMI)[S4], Adjusted Mutual Information (AMI)[S5]) for each pair of state assignments obtained in the 50 trials. A score of 1 using any of these metrics indicates that the state assignments have been the same. By contrast, lower scores indicate more dissimilarity in state assignments between trials. We then obtained a distribution for each type of VAMPnet input (shown as violin plots in Figure S5). The plot shows that, regardless of the scoring metric, the mean of each distribution increases as the dimensionality of the input features increases, indicating an increase in consistency of state assignments. It is also clear from the plot that when all backbone contacts were used as VAMPnet input, at least two sets of state assignments were obtained, as indicated by the bimodal distribution of the scores. One out of the 50 trials with compressed features ($n = 150$ and $n = 500$) was an outlier, as is also clear from Figure 3a of the main text.

Changing the architecture of VAMPnet networks for each set of input descriptors. In the previous sections and in the main text, the size of the VAMPnet hidden layers was kept constant to keep the comparison consistent. In Figure S6, we show the results for the case when the architecture of VAMPnet is adjusted for each input dimension. The architecture of VAMPnet used for each input dimension is shown in Table S2. The figure shows that these larger networks did not significantly improve the results discussed in the main text. It is important to note that when such large networks are used to train VAMPnet, the number of model parameters is significantly larger than the amount of available training data.

Random feature selection instead of random compression. To verify if random compression using neural networks is indeed necessary, we repeated the calculations for NTL9 by replacing the compression networks that generate n input features with randomly selected n features. In Figure S7, we show a plot quite similar to the one shown in the main text Figure 3. Here, n-dimensional feature sets were generated by randomly selecting n backbone contacts from the complete set of 6786 backbone contacts. For each dimension n, we performed 25 trials with 25 different sets of randomly selected contacts. This example shows that even with 500 randomly selected features, it was not possible to obtain consistent clusters. Not only does the population of each cluster show significant variability, but the

cluster assignments are also significantly less uniform than those shown in the main text Figure 3.

1.2.2 TICA and PCA

We compared the two slowest TICA (and PCA) eigenvalues obtained when either all Cα distances or compressed features were used as input. At first, we obtained the 2 TICA (and PCA) components using all Cα distances as input. We then obtained 2 TICA (and PCA) components using compressed features of different dimensionalities (2, 6, 10, 15, 20, 35, 50, 100, 150, 200, 250, 300). For a given dimensionality, we generated 25 different sets of compressed features to obtain 25 sets of TICA (and PCA) components. The time lag used for TICA is 50 ns.

We then measured the canonical cross-correlations between the TICA (or PCA) components obtained in a given trial and those obtained using all 741 Cα distances as input. The distribution of the cross-correlations obtained for a given compressed feature dimension is shown in Figure S8a. We observed that with a compressed dimension $n \geq 100$, a high correlation was obtained between the 2 PCA components using compressed features and those obtained using all 741 distances across the 25 trials. As few as 20 dimensions of compressed features were sufficient to obtain the first principal component, while at least 100 dimensions were necessary to obtain a more correlated second component. Although the first TICA component was recovered with just 20-dimensional compressed features, the second TICA component obtained using compressed features had very low correlation with the one obtained when all distances were used as input. To illustrate how the projections with high and low mean cross-correlations appear, we show selected PCA and TICA projections in Figures S8b and S8c.

1.3 Villin (2f4k) at 360K

We trained multiple VAMPnet models with different input features to obtain a 4-state decomposition of the 300 μs MD trajectory of the double-norleucin villin variant (2f4k),[S6] referred to as "villin" in the following. In addition to using all backbone contacts, as in the NTL9 case, we also used all positions, Cα contacts, and maximum-gap shifted backbone dihedral angles[S7]. For each of these input types, we used different VAMPnet architectures as listed in Table S3. We then obtained 25 sets of compressed features of different dimensionalities created using random nonlinear transformations of either of the four input types. In Figures S9 and S10, we show the three slowest timescales obtained using each set of input and the mean fraction of native contacts. Yet again, it is clear from the plots that the clusters tend to be more consistent across different trials when the dimensionality of the compressed features increases. We also show the similarity score distributions obtained for each set of inputs in Figure S11. While the state assignments become more consistent with increased compressed feature dimensionality, a bimodal distribution is observed across different input feature types. This bimodality across input feature types is another indicator of the existence of more than one way to cluster the villin trajectory into four states.

2 SI Tables

Table S1: Architecture of VAMPnet lobes for NTL9.

Architecture of VAMPnet lobes for the different cases							
Case	Input	Hidden 1	Hidden 2	Hidden 3	Hidden 4	Hidden 5	Output
All backbone contacts	6786	41	25	15	9	6	5
Compressed	500	41	25	15	9	6	5
Compressed	200	41	25	15	9	6	5
Compressed	150	41	25	15	9	6	5
Compressed	100	41	25	15	9	6	5
Compressed	50	41	25	15	9	6	5
Compressed	30	21	15	11	8	6	5

Table S2: Varying architectures of VAMPnet lobes for NTL9.

Architecture of VAMPnet lobes for the different cases							
Case	Input	Hidden 1	Hidden 2	Hidden 3	Hidden 4	Hidden 5	Output
All backbone contacts	6786	882	198	45	11	3	5
Compressed	500	200	80	32	13	6	5
Compressed	200	96	46	22	11	6	5
Compressed	150	76	39	20	11	6	5
Compressed	100	55	31	18	10	6	5
Compressed	50	32	21	14	9	6	5
Compressed	30	21	15	11	8	6	5

Table S3: Architecture of VAMPnet lobes for double-norleucin variant of villin headpiece.

Architecture of VAMPnet lobes with the different inputs for villin					
Case	Input	Hidden 1	Hidden 2	Hidden 3	Output
All backbone contacts	5460	107	21	5	4
All positions	1731	229	31	5	4
All $c\alpha$ contacts	595	113	22	5	4
All angles	66	26	11	5	4

3 SI Figures

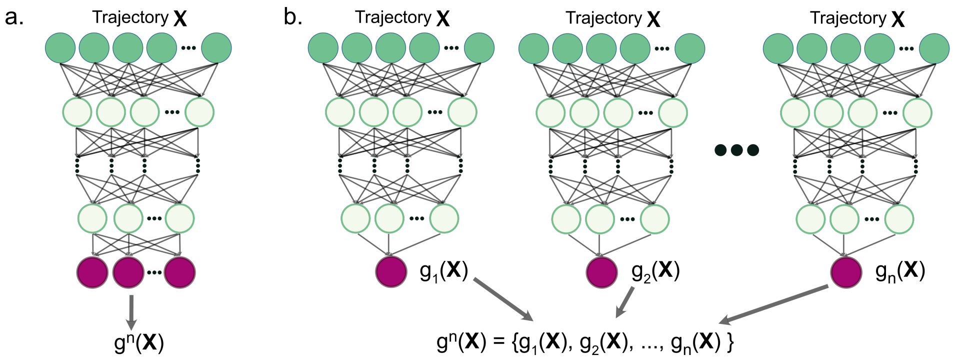

Figure S1: Two different approaches for obtained randomly compressed features. (a)n-dimensional compression is generated using only one neural network with n output neurons. (b) n-dimensional compression is generated using n different networks, each with one output neuron.

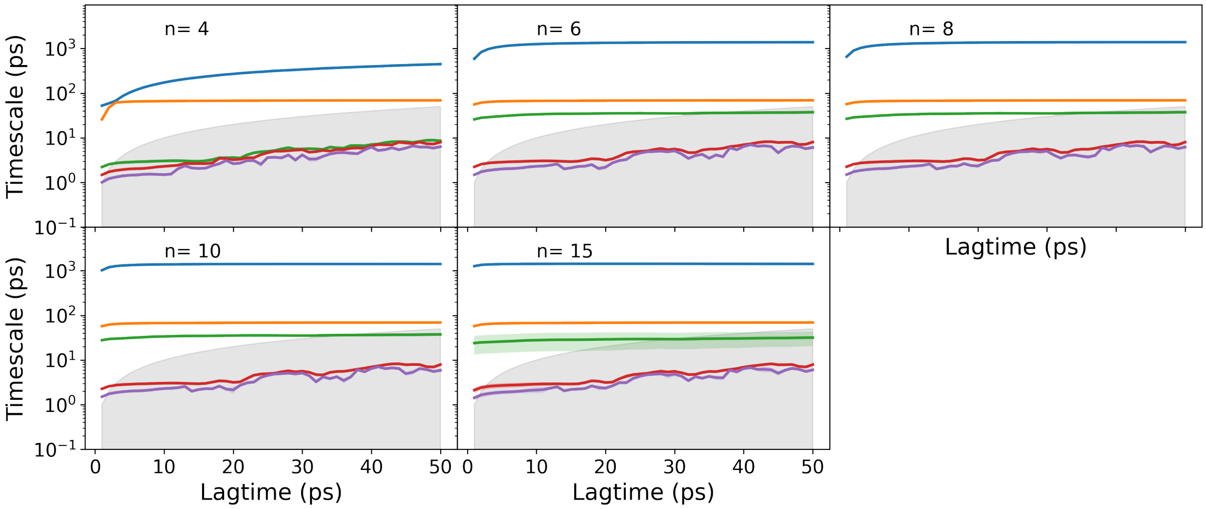

Figure S2: Implied timescales for alanine dipeptide obtained from VAMPnet using different numbers of PCA components as input. The mean timescale across 25 trials are shown in thick lines while the shaded regions indicate the standard deviation. The regime where the estimated timescale drops below the lagtime is indicated using the grey shaded areas.

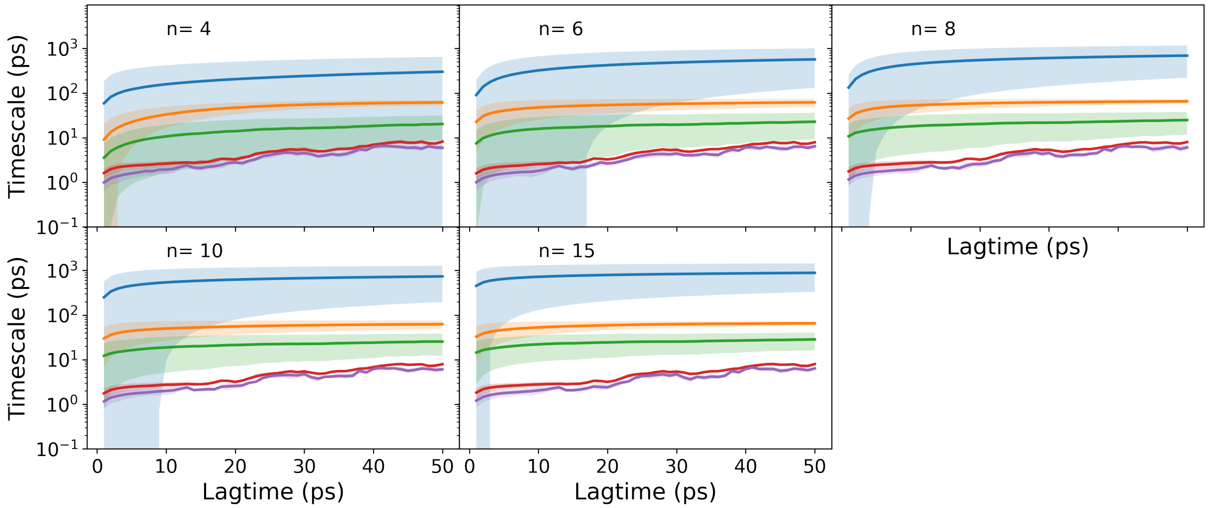

Figure S3: Implied timescales obtained from VAMPnet using compressed features as input. Each set of n-dimensional compressed feature was generated using a neural network with n outputs. The mean timescale across 25 trials are shown in thick lines while the shaded regions indicate the standard deviation.

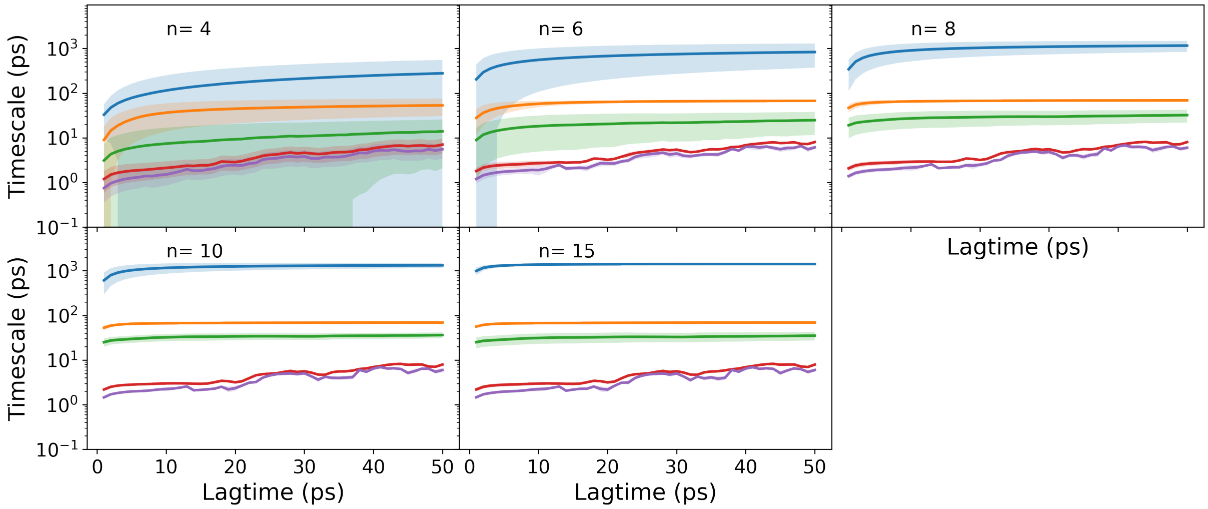

Figure S4: Implied timescales obtained from VAMPnet using compressed features as input. Each set of n-dimensional compressed feature was generated using n neural networks with one output. The mean timescale across 25 trials are shown in thick lines while the shaded regions indicate the standard deviation.

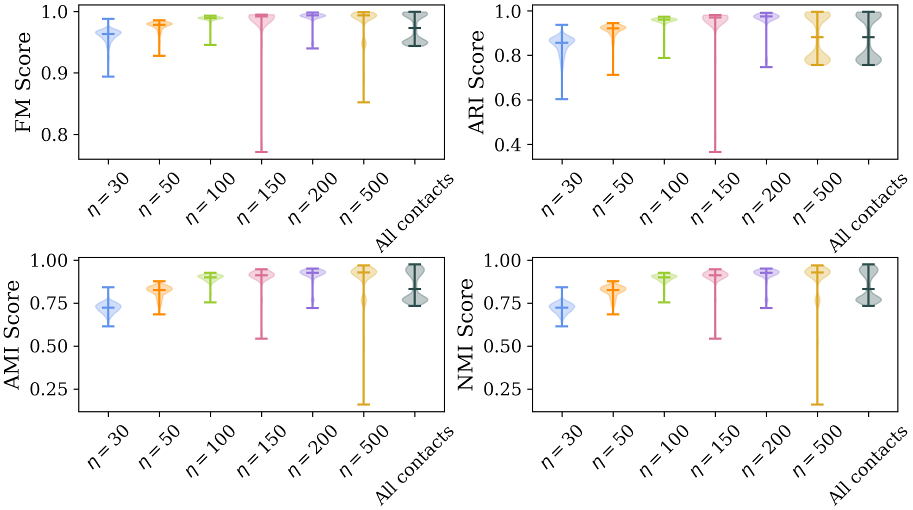

Figure S5: Distribution of similarity scores for state assignments obtained using different VAMPnet inputs for the NTL9 protein. Results are shown for the similarity metrics Fowlkes-Mallows Score (FM), Adjusted Rand Index (ARI), Adjusted Mutual Information(AMI), Normalized Mutual Information (NMI).

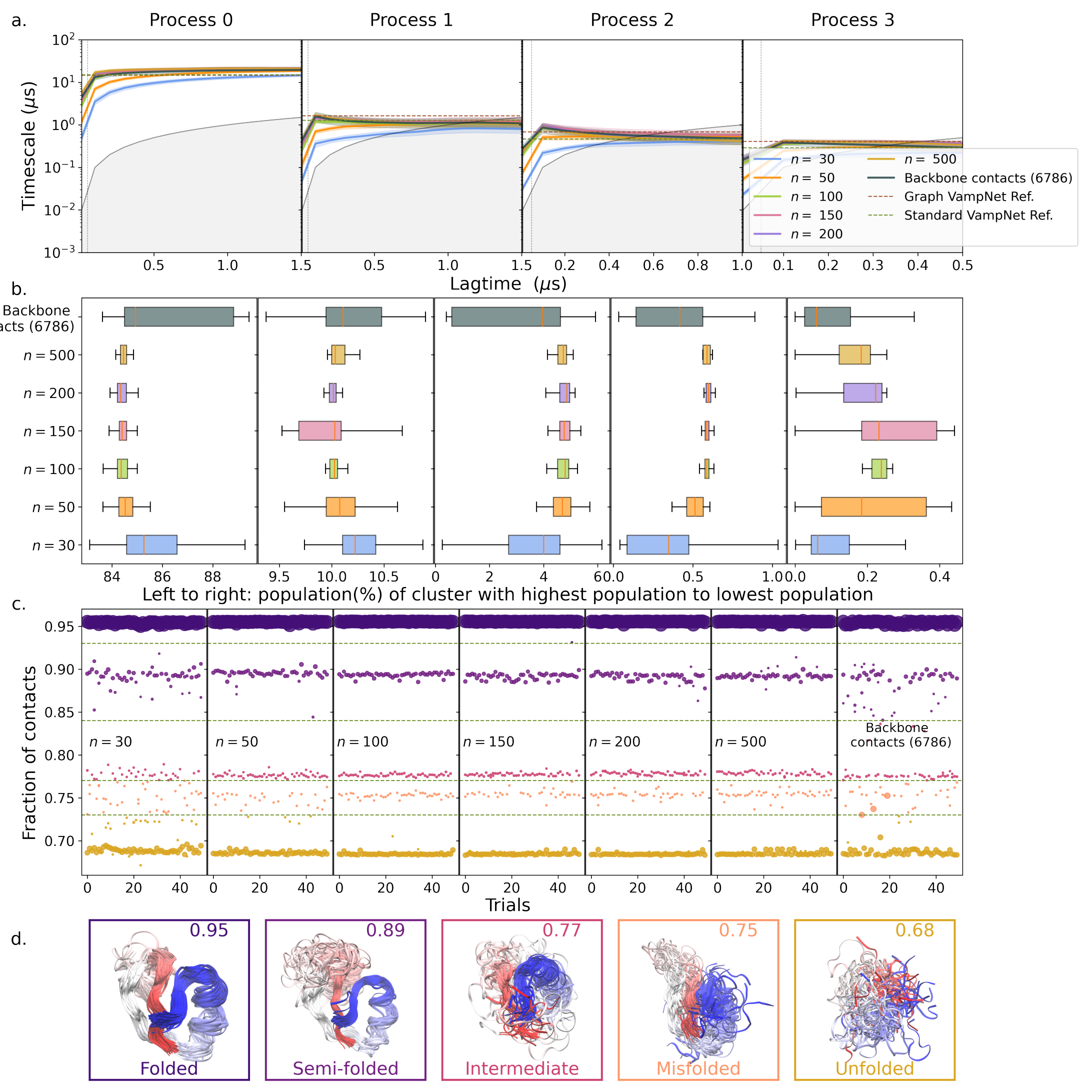

Figure S6: Larger networks used for VAMPNet. (a) Timescale of 4 slowest relaxation processes (left to right) extracted from 50 trials as function of lagtime, with fixed time lag of 50 ns for VAMPnet training (vertical dotted line). The dimension of the random projections, when used, are indicated by n in the legend. Backbone contacts (6786) indicate that no compression is used. "Graph VAMPnet" are the results of Ghorbani et al.[S8], and "Standard VAMPnet" those of Mardt et al.[S1]. The gray area indicates timescales less than the lagtime. (b) Population of the five clusters obtained. Left to Right: Most populated cluster in each trial to least populated. (c) Mean fraction of native contacts for the five clusters obtained in the 50 trials for each method, as indicated in each subpanel. Colors correspond to the clusters in d. The size of each dot is proportional to cluster population. The four dashed lines at contact fractions of 0.93, 0.84, 0.77, and 0.73 indicate boundaries between different cluster structures. (d) Backbone structures representative of the clusters with different fractions of native contacts in c. The clustering was obtained in one of the trials with $n = 100$ random projections from the set of results shown in the main text. The colors of the surrounding boxes correspond to the color of the cluster in c. The value of the mean fraction of native contacts in each cluster is shown on top for reference.

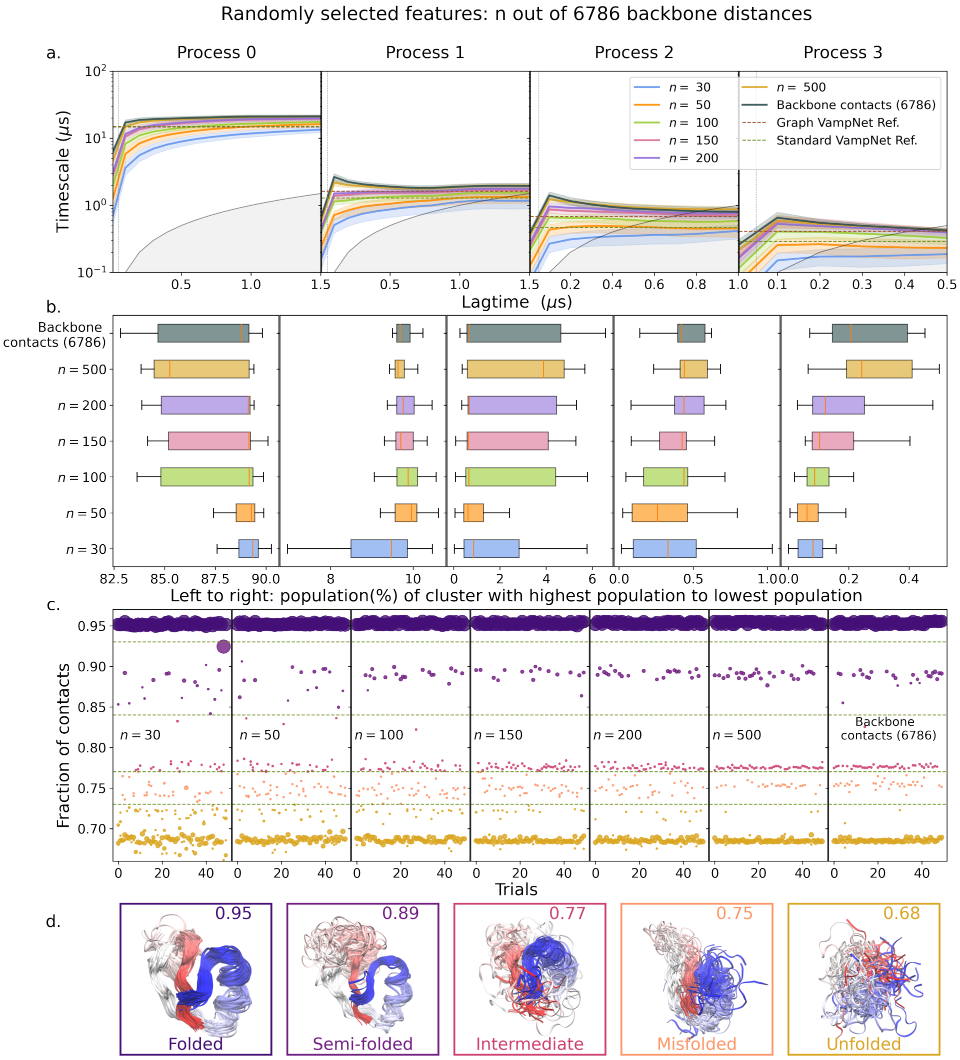

Figure S7: Randomly selected backbone contacts in NTL9 used as input for VAMPNet. Compare to Figure 3 in the main text, in which the results of random compression are shown. (a) Timescale of 4 slowest relaxation processes (left to right) extracted from 50 trials as function of lagtime, with fixed time lag of 50 ns for VAMPnet training (vertical dotted line). The dimension of the random projections, when used, are indicated by n in the legend. Backbone contacts (6786) indicate that no compression is used. "Graph VAMPnet" are the results of Ghorbani et al.[S8], and "Standard VAMPnet" those of Mardt et al.[S1]. The gray area indicates timescales less than the lagtime. (b) Population of the five clusters obtained. Left to Right: Most populated cluster in each trial to least populated. (c) Mean fraction of native contacts for the five clusters obtained in the 50 trial for each method, as indicated in each subpanel. Colors correspond to the clusters in d. The size of each dot is proportional to cluster population. The four dashed lines at contact fractions of 0.93, 0.84, 0.77, and 0.73 indicate boundaries between different cluster structures. (d) Backbone structures representative of the clusters with different fractions of native contacts in c. The clustering was obtained in one of the trials with $n = 100$ random projections from the set of results shown in the main text. The colors of the surrounding boxes correspond to the color of the cluster in c. The value of the mean fraction of native contacts in each cluster is shown on top for reference.

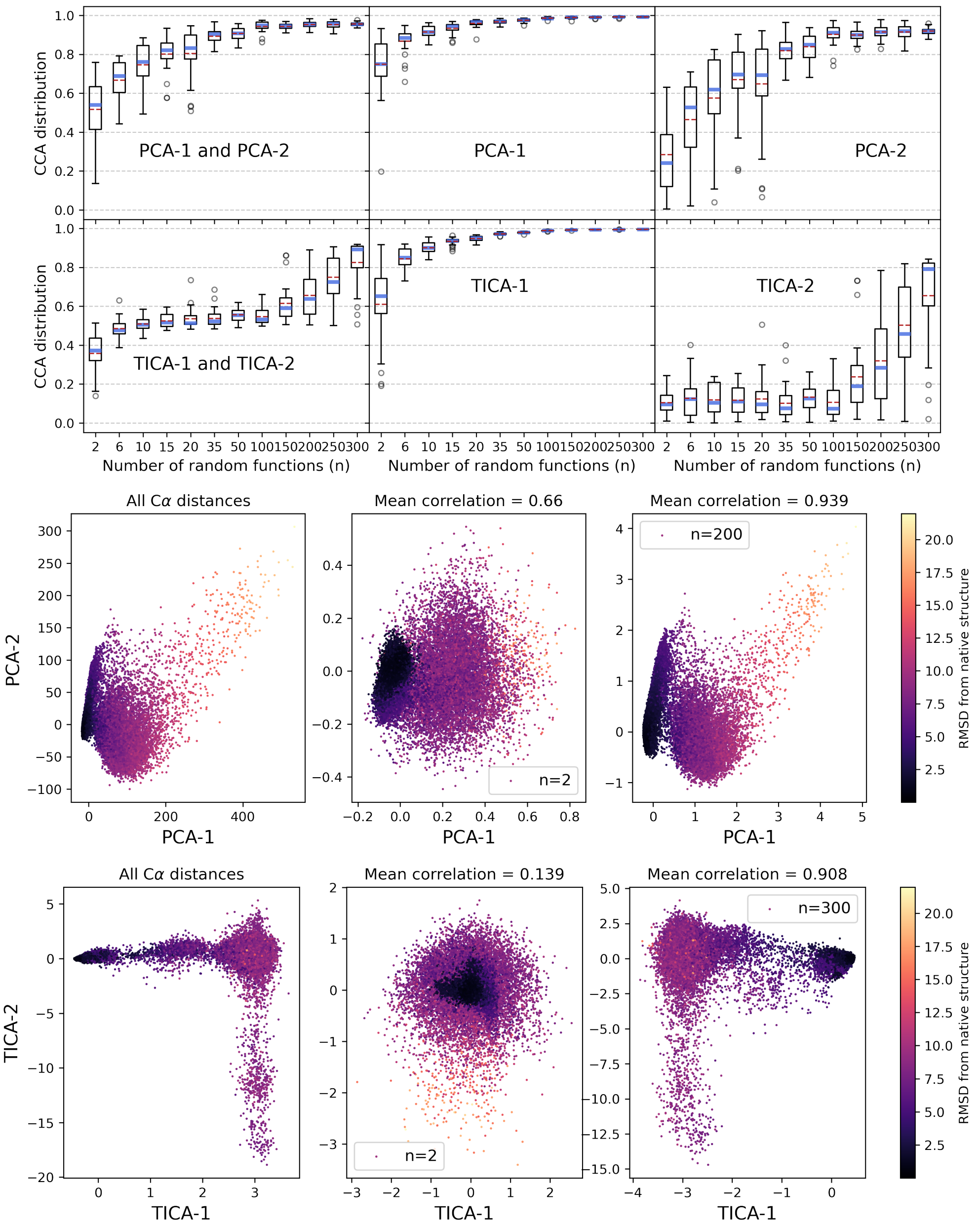

Figure S8: TICA and PCA components obtained for NTL9 using all Cα distances or compressed features created using all Cα distances. (a) Distribution of cross correlations. (b) Examples of PCA projections with all 741 Cα distances as input (left), compressed features of dimension 2 (middle), compressed features of dimension 200 (right). The color of data indicates the RMSD from the native folded structure with darker color indicated more folded structures. (c) Examples of TICA projections with all 741 Cα distances as input (left), compressed features of dimension 2 (middle), compressed features of dimension 200 (right). The color of data indicates the RMSD from the native folded structure with darker color indicated more folded structures.

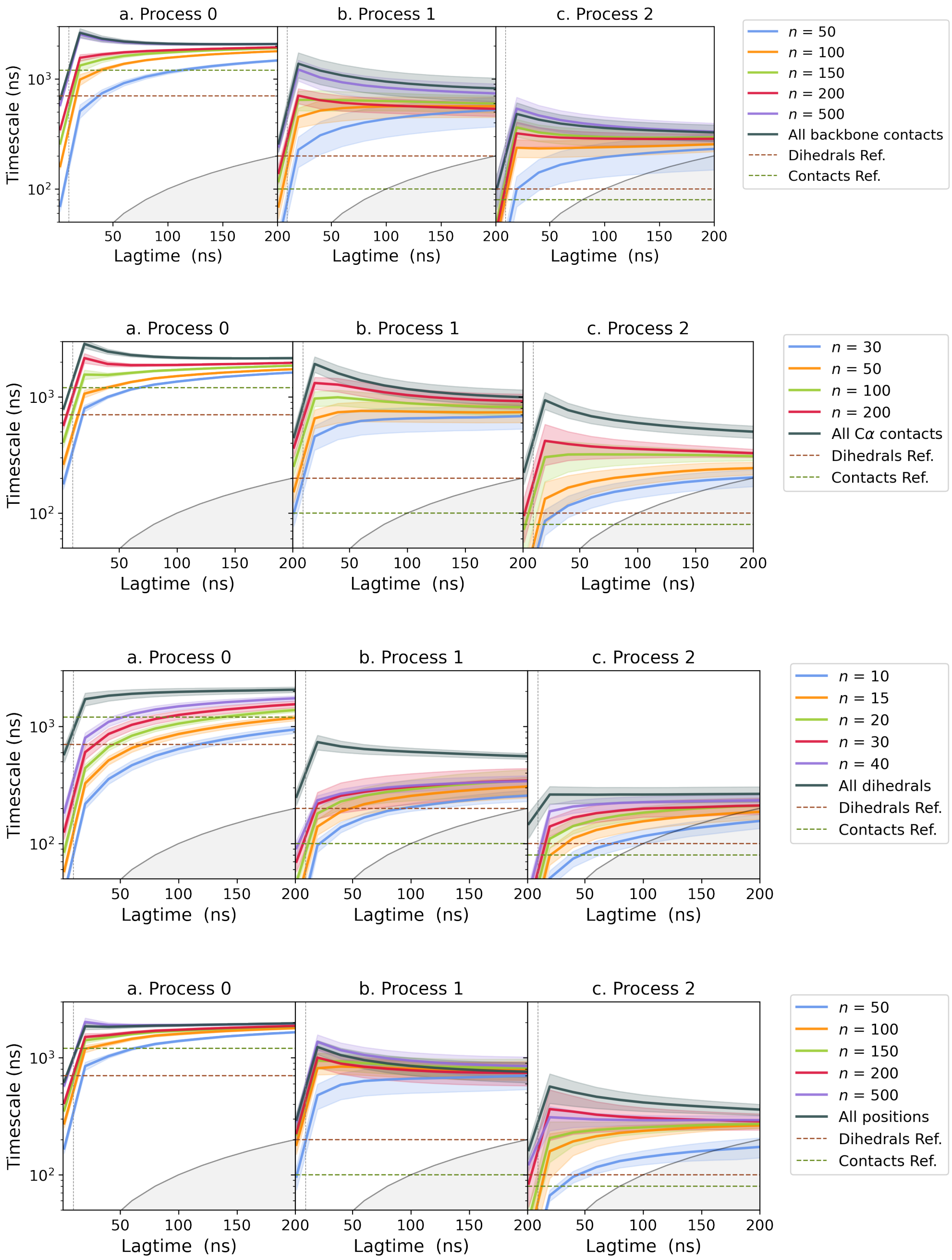

Figure S9: Results for the double-norleucin variant of villin. The three implied timescales were obtained using different input features for VAMPnet, as indicated in the legend. For reference, two sets of timescales reported by Nagel et al.[S7] are shown as dashed lines (brown: dihedrals, green: contacts). As input features, we used (a) backbone contacts, (b) Cα contacts, (c) dihedral angles, (d) the positions of all atoms after rigid-body superposition.

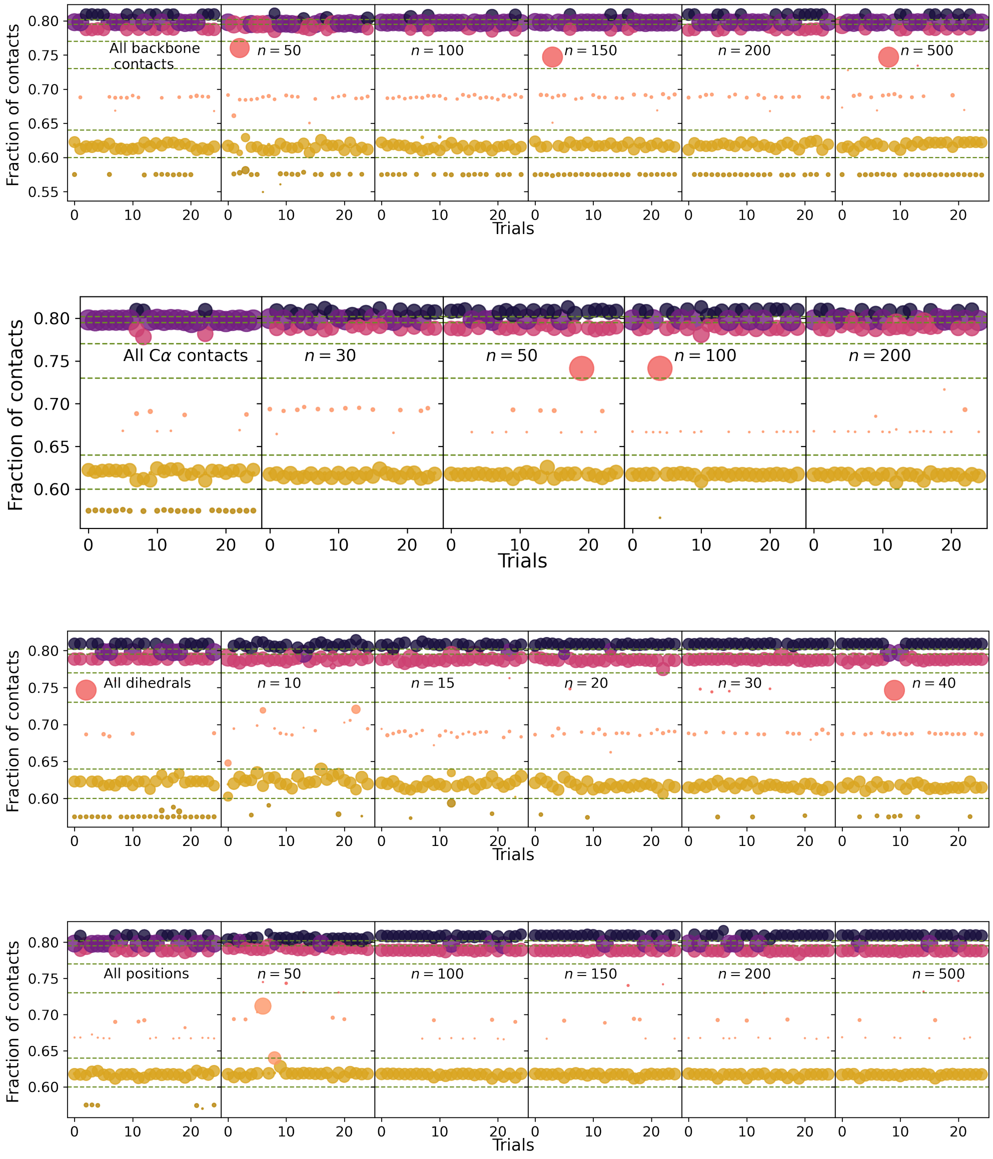

Figure S10: Results for the double-norleucin variant of villin. The mean fraction of native contacts for the clusters across multiple trials and different input features is shown. Colors correspond to regrouped clusters obtained by partitioning the VAMPnet state assignments into 7 sets. The six horizontal dashed lines at contact fractions of 0.802, 0.795, 0.77, 0.73, 0.64, and 0.6 indicate boundaries used for regrouping. The size of each dot is proportional to cluster population.

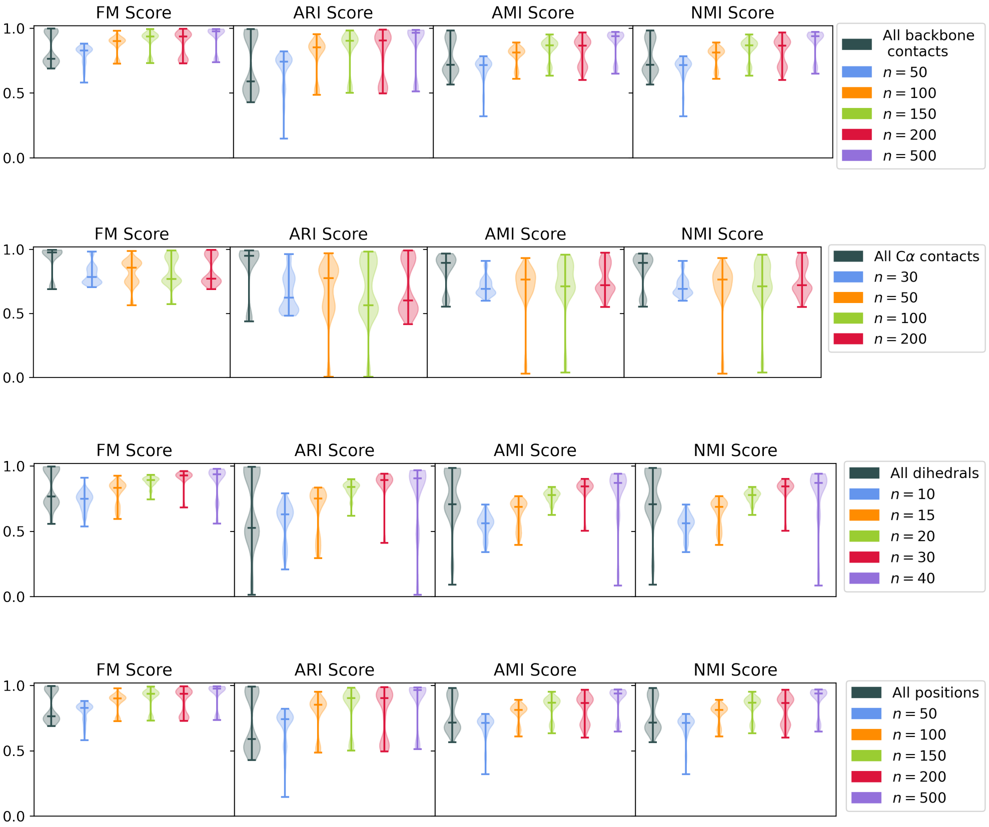

Figure S11: Distribution of similarity scores for state assignments obtained using different VAMPnet inputs for the Villin trajectory. Results are shown for the similarity metrics Fowlkes-Mallows Score (FM), Adjusted Rand Index (ARI), Adjusted Mutual Information(AMI), Normalized Mutual Information (NMI).

4 SI References

References

(S1) Mardt, A.; Pasquali, L.; Wu, H.; Noé, F. VAMPnets for Deep Learning of Molecular Kinetics. *Nature Communications* **2018**, *9*, 5.

(S2) Fowlkes, E. B.; Mallows, C. L. A Method for Comparing Two Hierarchical Clusterings. *Journal of the American Statistical Association* **1983**, *78*, 553–569.

(S3) Hubert, L.; Arabie, P. Comparing Partitions. *Journal of Classification* **1985**, *2*, 193–218.

(S4) Strehl, A.; Ghosh, J. Cluster Ensembles — A Knowledge Reuse Framework for Combining Multiple Partitions. *Journal of Machine Learning Research* **2003**, *3*, 583–617.

(S5) Vinh, N. X.; Epps, J.; Bailey, J. Information Theoretic Measures for Clusterings Comparison: Variants, Properties, Normalization and Correction for Chance. *Journal of Machine Learning Research* **2010**, *11*, 2837–2854.

(S6) Lindorff-Larsen, K.; Piana, S.; Dror, R. O.; Shaw, D. E. How Fast-Folding Proteins Fold. *Science* **2011**, *334*, 517–520.

(S7) Nagel, D.; Sartore, S.; Stock, G. Selecting Features for Markov Modeling: A Case Study on HP35. *Journal of Chemical Theory and Computation* **2023**, *19*, 3391–3405.

(S8) Ghorbani, M.; Prasad, S.; Klauda, J. B.; Brooks, B. R. GraphVAMPNet, Using Graph Neural Networks and Variational Approach to Markov Processes for Dynamical Modeling of Biomolecules. *The Journal of Chemical Physics* **2022**, *156*, 184103.
